# Effect of dapagliflozin on heart failure with reduced ejection fraction in children

**DOI:** 10.3389/fcvm.2026.1785458

**Published:** 2026-04-17

**Authors:** Shaoyong Lin, Yiwei Chen, Diqi Zhu, Jinjin Wu, Lijun Fu, Fen Li, Xiaofeng Guo

**Affiliations:** 1 Department of Cardiology, Fujian Children’s Hospital (Fujian Branch of Shanghai Children’s Medical Center), College of Clinical Medicine for Obstetrics & Gynecology and Pediatrics, Fujian Medical University, Fuzhou, Fujian, China; 2 Department of Cardiology, Shanghai Children’s Medical Center Affiliated to Shanghai Jiao Tong University School of Medicine, Shanghai, China

**Keywords:** children, dapagliflozin, effect, heart failure with reduced ejection fraction, sodium-glucose co-transporter protein-2 inhibitor

## Abstract

**Objective:**

To explore the efficacy and safety of dapagliflozin therapy in children with heart failure with reduced ejection fraction.

**Methods:**

This was a retrospective cohort study based on electronic medical records. For the retrospective cohort study, 32 children with heart failure with reduced ejection fraction diagnosed at Shanghai Children's Medical Center and Fujian Children's Hospital from June 2021 to June 2023 and who were treated with dapagliflozin were enrolled. For pediatric patients weighing <10 kg, dapagliflozin was administered at a dosage of 0.2 mg/kg/d. Patients with a body weight ranging from 10 kg to less than 20 kg (20 > weight ≥10 kg) received dapagliflozin at a dose of 2.5 mg/d. Those weighing between 20 kg and less than 30 kg (30 > weight ≥20 kg) were prescribed dapagliflozin at 5 mg/d. For patients weighing 30 kg or more, the initial dose of dapagliflozin was 5 mg/d, which was increased to 10 mg/d after one month. 42 children treated during the same period without dapagliflozin were included as the control group. All children were treated with standard guideline-directed triple combination of ACEI/ARNI, *β*-blocker, and aldosterone receptor antagonist. Clinical characteristics, underlying diseases, cardiac function ratings, left ventricular ejection fraction, B-type natriuretic peptide, and blood biochemical indexes were collected. Changes in cardiac function ratings, left ventricular ejection fraction, and B-type natriuretic peptide at 24 (±2) weeks of treatment were observed; the incidence of hospitalization or death due to exacerbation of heart failure at 24 (±2) weeks of treatment was observed secondarily. Adverse medicine events such as hypoglycemia or hypotension were also observed during treatment.

**Results:**

Among the 32 children, 20 were male and 12 were female, aged (6.32 ± 4.00) years; weight (22.19 ± 12.66) kg. The underlying diseases included dilated cardiomyopathy in 25 cases, dilated cardiomyopathy combined with complete left bundle branch block in two cases, postoperative congenital heart disease in four cases, and atrial tachycardia in one case. At 24 (±2) weeks of treatment, left ventricular ejection fraction level significantly increased compared with baseline (*P* < 0.001); B-type natriuretic peptide decreased (*P* < 0.001); and New York Heart Association (NYHA) cardiac function ratings improved significantly (*P* = 0.034). Compared with the control group, children in the dapagliflozin group showed greater improvement in left ventricular ejection fraction (*P* = 0.032) and NYHA cardiac function rating (*P* = 0.038), whereas no significant difference was observed in BNP levels (*P* = 0.071). Three children developed urinary tract infection during the administration of the medication, and three children developed hypotension, while no other adverse reactions, such as hypoglycemia or hepatic or renal function impairment, were observed.

**Conclusion:**

Dapagliflozin was associated with improvements in left ventricular ejection fraction, BNP, and NYHA cardiac function classification in children with heart failure with reduced ejection fraction. No hospitalization or death due to the deterioration of heart failure and no serious adverse reactions were observed. The treatment was generally well tolerated, and the weight-stratified dosing strategy showed acceptable short-term safety.

## 1 Introduction

Sodium-glucose co-transporter protein-2 inhibitors (SGLT2i) have been shown to reduce the incidence of hospitalization for exacerbations and cardiovascular event mortality in adult chronic cardiac failure (CHF) patients in numerous clinical studies and has been recommended as one of the four cornerstone pharmacological therapies for the treatment of CHF by several guidelines (1–3). Dapagliflozin, a classic representative of SGLT2i, has been widely used in the treatment of adult CHF, but few studies have addressed its efficacy and safety in pediatric CHF patients (4–6). CHF in children is often complicated by various types of cardiovascular diseases, including congenital heart disease, cardiomyopathy, myocarditis, and arrhythmia, of which heart failure with reduced ejection fraction (HFrEF) represents an important clinical subtype in clinical practice (7–9). If the efficacy and safety of dapagliflozin can be verified in pediatric HFrEF cases, it will undoubtedly bring new hope for the treatment of such pediatric cases. The aim of this study was to evaluate the efficacy and safety of dapagliflozin in children with HFrEF and to explore safe dosing of dapagliflozin in children with HFrEF.

## 2 Materials and methods

### 2.1 Materials

A retrospective cohort study was used. Clinical data were extracted from electronic medical records. A total of 32 children with HFrEF who attended the Department of Cardiology of Shanghai Children's Medical Center and Fujian Children's Hospital from June 2021 to June 2023 and met the eligibility criteria were included as the study group. The eligibility criteria were (1) aged from 6 months to 18 years; (2) having received angiotensin converting enzyme inhibitor (ACEI) or angiotensin receptor neprilysin inhibitor (ARNI), beta-blocker, or aldosterone receptor antagonist for at least 2 weeks but less than 4 weeks, had a two-dimensional echocardiogram that indicates a left ventricular ejection fraction (LVEF) of less than 40%, and had a New York Heart Association (NYHA) functional classification between I and III; and (3) estimated glomerular filtration rate (eGFR) ≥ 60 mL/(min·1.73 m^2^). The exclusion criteria were (1) clinical assessment of children in NYHA class IV status; (2) children with conditions requiring vasoactive drug therapy; (3) children with blood pressure lower than P5 in children of the same sex and age; (4) children with any type of diabetes mellitus; (5) comorbidity with uncontrolled urinary tract infections; (6) comorbidity with other medical conditions such as hepatic and renal function abnormalities that may affect drug absorption, distribution, metabolism, or excretion; or (7) discontinuation of dapagliflozin during follow-up due to severe complications resulting in incomplete follow-up. Dapagliflozin was prescribed as off-label therapy as part of routine clinical care, and written informed consent for off-label drug use was obtained from the legal guardians according to institutional clinical practice. 42 children treated during the same period who received standard heart failure therapy without dapagliflozin were included as the control group.

### 2.2 Methods

#### 2.2.1 Collect and analyze the children's clinical data

The children's age, weight, gender, height, body mass index, blood pressure, clinical symptoms, NYHA cardiac function class, LVEF, type B natriuretic peptide (BNP), blood glucose, liver and kidney function, blood electrolytes, glomerular filtration rate, and so on were collected and analyzed. The baseline indexes before treatment were reviewed after receiving dapagliflozin treatment for 3 days, 1 week, 2 weeks, and 1 month, and followed up to 24 (±2) weeks month by month.

#### 2.2.2 Treatment regimen

All patients received guideline-directed triple therapy, including an ACEI/ARNI, a beta-blocker, and an aldosterone receptor antagonist, and doses were up-titrated as tolerated according to clinical condition. Patients were assigned to the control group if their guardians refused dapagliflozin because of concerns regarding its off-label use and potential risks. In addition, children in the dapagliflozin group were treated with dapagliflozin according to a weight-stratified dosing regimen. For pediatric patients weighing <10 kg, dapagliflozin was administered at a dosage of 0.2 mg/kg/d. Patients with a body weight ranging from 10 kg to less than 20 kg (20 > weight ≥10 kg) received dapagliflozin at a dose of 2.5 mg/d. Those weighing between 20 kg and less than 30 kg (30 > weight ≥20 kg) were prescribed dapagliflozin at 5 mg/d. For patients weighing 30 kg or more, the initial dose of dapagliflozin was 5 mg/d, which was increased to 10 mg/d after one month.

#### 2.2.3 Observation indexes

The main observation indexes were the changes of NYHA cardiac function class, LVEF, and BNP when receiving dapagliflozin treatment for 24 (±2) weeks. The secondary observation indexes were the occurrence of hospitalization or death due to exacerbation of CHF within 24 (±2) weeks of dapagliflozin treatment. Adverse medicine events such as hypoglycemia or hypotension were also observed during treatment.

#### 2.2.4 Statistical methods

Statistical analyses were conducted using SPSS 27.0 (IBM Corp.). Continuous variables with normal distribution were expressed as mean ± standard deviation (SD) and non-normally distributed data were presented as median (interquartile range, IQR). Within-group comparisons were performed using paired t-tests or Wilcoxon signed-rank tests as appropriate. Between-group comparisons were conducted using independent samples *t*-tests or Mann–Whitney *U*-tests. Categorical variables were summarized as frequencies (%) and evaluated through Chi-square test or Fisher's exact test as appropriate. Normality was assessed using Shapiro–Wilk tests. A two-tailed *P*-value < 0.05 defined statistical significance.

## 3 Results

### 3.1 Basic information

**Figure 1:**
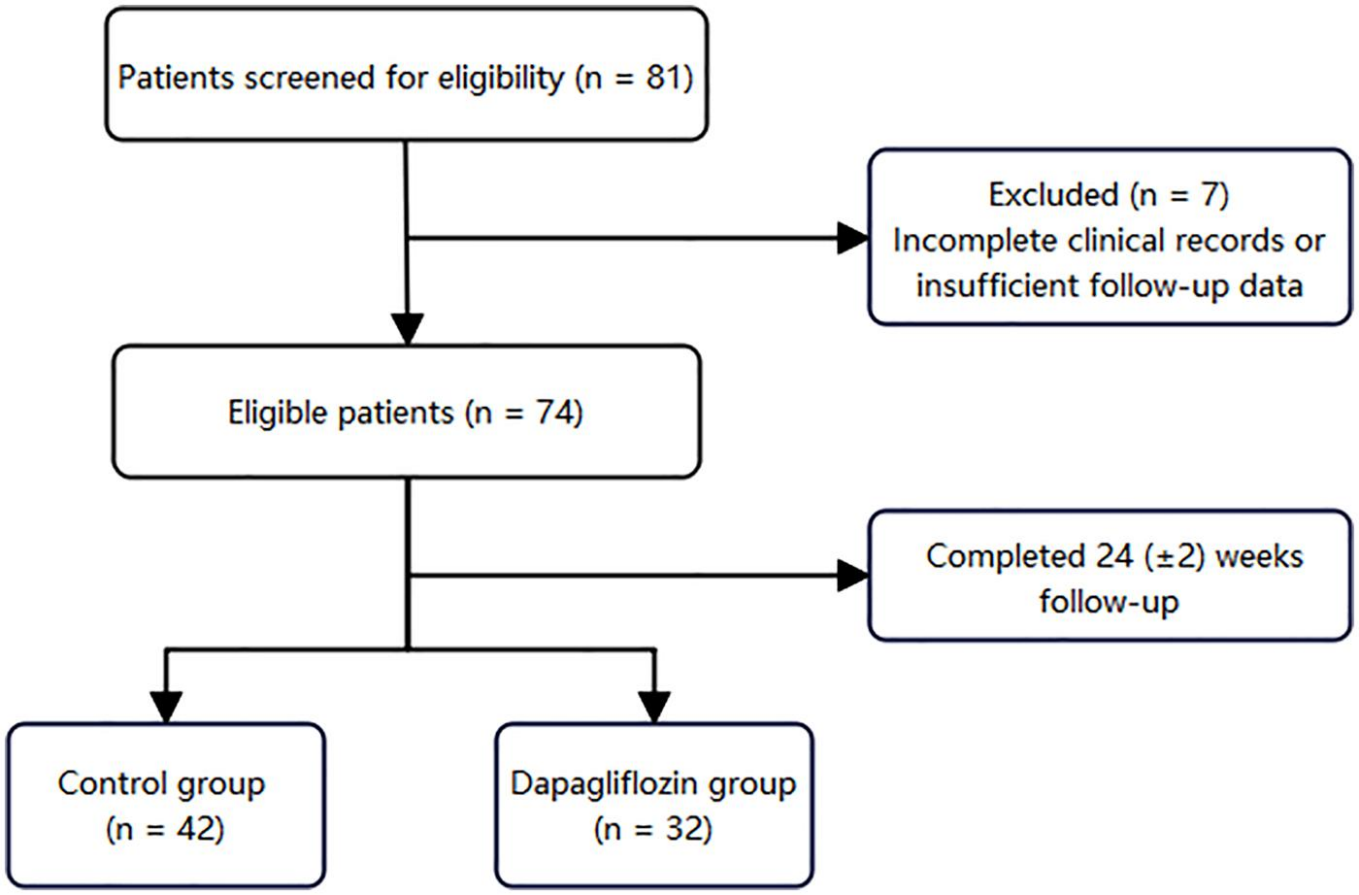
STROBE flow diagram of patient enrollment.

A total of 81 children were screened to meet the eligibility criteria, of which seven cases were excluded due to incomplete clinical records or insufficient follow-up data. Therefore, 74 children were included in the analysis, including 32 children in the dapagliflozin group and 42 children in the control group. All children completed the 24 (±2) week follow-up requirement (Figure 1).

In the dapagliflozin group, there were 20 males and 12 females, age (6.32 ± 4.00) years. The minimum age was 9 months and the maximum age was 14 years; body weight (22.19 ± 12.66) kg ranged from 7.1 kg to 60 kg; LVEF was (32.15 ± 4.87)%, and BNP was 721.00pg/mL(IQR:386.25–1,965.50). The children's underlying diseases included dilated cardiomyopathy in 25 cases, dilated cardiomyopathy combined with complete left bundle branch block in two cases, heart-spleen syndrome after GLENN in two cases, pulmonary artery atresia with ventricular septal defect after radical surgery in one case, valvular cardiomyopathy after mitral valve mechanical valve replacement in one case, and atrial tachycardia in one case (Table 1). Of the 32 cases, 21 cases had received sacubitril valsartan 3.2 mg/kg/d or up to 100 mg/d before dapagliflozin therapy, 11 received enalapril 0.1 to 0.2 mg/kg/d, and all children received beta-blockers (metoprolol 1–1.5 mg/kg/d, maximum dose 50 mg) and aldosterone receptor antagonists (spironolactone 2 mg/kg/d, maximum dose 40 mg) prior to the initiation of dapagliflozin. 11 children also received digoxin 6–10ug/kg/d, two children with dilated cardiomyopathy combined with complete left bundle branch block received cardiac resynchronization therapy during follow-up, and one child with paroxysmal atrial tachycardia underwent radiofrequency ablation at the end of the final follow-up.

**Table 1:** Basic information. Comparison of baseline data between the dapagliflozin group and the control group.

Variable	dapagliflozin group (*n* = 32)	control group (*n* = 42)
Gender [n (%)]		
Male	20 (62.5)	21 (50)
Female	12 (37.5)	21 (50)
Age (years)	6.32 ± 4.00	4.75 ± 3.29
Weight (kg)	22.19 ± 12.66	17.87 ± 10.19
LVEF (%)	32.15 ± 4.87	31.64 ± 4.05
BNP (pg/mL)	721.00 (386.25–1,965.50)	978.50 (653.50–1,844.75)
NYHA Cardiac Function Classification [n (%)]		
Class I	11 (34.4)	12 (28.6)
Class II	16 (50)	19 (45.2)
Class III	5 (15.6)	11 (26.2)
Underlying diseases (n)		
DCM	25	28
DCM combined with LRBBB	2	1
Post-operative congenital heart disease	3	6
Post-operative mechanical valve replacement	1	2
preexcitation syndrome	0	4
atrial tachycardia	1	1

LVEF, left ventricular ejection fraction; BNP, B-type natriuretic peptide; NYHA, New York Heart Association; DCM, dilated cardiomyopathy; LRBBB, complete left bundle branch block.

In the control group, there were 21 males and 21 females, age (4.75 ± 3.29) years, weight (17.87 ± 10.19) kg. There was no significant difference in age (*P* = 0.068) or weight (*P* = 0.112) between the two groups. Baseline LVEF was (31.64 ± 4.05)% and BNP was 978.50pg/mL (IQR:653.50–1,844.75), with no significant difference in LVEF (*P* = 0.619) or BNP (*P* = 0.147) between the two groups. There was also no significant difference in the baseline NYHA cardiac function classification between the two groups (*P* = 0.566). In the control group, there were 28 cases of dilated cardiomyopathy, one case of dilated cardiomyopathy combined with complete left bundle branch block, six cases of various types of postoperative congenital heart disease, two cases of valvular cardiomyopathy after tricuspid mechanical valve replacement, four cases of preexcitation syndrome, and one case of atrial tachycardia. In the control group, the children had been treated with ACEI/ARNI, beta-blockers, and aldosterone receptor antagonists for at least 2 weeks but less than 4 weeks at baseline. Additionally, 12 children were concomitantly treated with digoxin, one child with dilated cardiomyopathy combined with complete left bundle-branch block was treated with cardiac resynchronization at approximately 12 weeks during follow-up, and four children with pre-excitation syndrome and one child with atrial tachycardia received radiofrequency ablation between 4 and 10 weeks during follow-up.

### 3.2 Observation indicators

The main observational indicators were that, for children in the dapagliflozin group at 24 (±2) weeks of treatment, LVEF significantly increased to (43.76 ± 7.58)% compared with baseline (*P* < 0.001); BNP significantly (*P* < 0.001) decreased to 315.00 pg/mL (IQR:193.15–626.25); and NYHA cardiac function rating improved significantly (*P* = 0.034) (Table 2).

**Table 2:** Observation indicators. Comparison of pre- and post-treatment data in the dapagliflozin group.

Variable	Before treatment (*n* = 32)	Post-treatment (*n* = 32)	*P*-value
LVEF (%)	32.15 ± 4.87	43.76 ± 7.58	<0.001
BNP (pg/mL)	721.00 (386.25–1,965.50)	315.00 (193.15–626.25)	<0.001
NYHA Cardiac Function Classification [n (%)]			0.034
Class I	11 (34.4)	21 (65.6)	-
Class II	16 (50)	10 (31.3)	-
Class III	5 (15.6)	1 (3.1)	-

LVEF, left ventricular ejection fraction; BNP, B-type natriuretic peptide; NYHA, New York Heart Association.

At 24 (±2) weeks of treatment, children in the control group similarly showed an increase in LVEF (39.68 ± 8.34)% (*P* < 0.001) from baseline; there was also a significant decrease in BNP 511.50pg/mL (IQR:308.75–859.75) (*P* < 0.001). However, there was no improvement in NYHA cardiac function ratings from baseline (*P* = 0.156) (Table 3).

**Table 3:** Observation indicators. Comparison of pre- and post-treatment data for the control group.

Variable	Before treatment (*n* = 42)	Post-treatment (*n* = 42)	*P*-value
LVEF (%)	31.64 ± 4.05	39.68 ± 8.34	<0.001
BNP (pg/mL)	978.50 (653.50- 1,844.75)	511.50 (308.75- 859.75)	<0.001
NYHA Cardiac Function Classification [n (%)]			0.156
Class I	12 (28.6)	15 (35.7)	-
Class II	19 (45.2)	23 (54.8)	-
Class III	11 (26.2)	4 (9.5)	-

LVEF, left ventricular ejection fraction; BNP, B-type natriuretic peptide; NYHA, New York Heart Association.

Compared with the control group, the dapagliflozin group showed greater improvement in LVEF (*P* = 0.032) and NYHA cardiac function rating (*P* = 0.038), whereas no difference was shown in BNP (*P* = 0.071).

Secondary observation indexes showed that, by 24 (± 2) weeks of follow-up, no heart failure deterioration requiring emergency hospitalization was observed in the dapagliflozin group. In the control group, three children developed worsening symptoms and signs such as poor appetite and swelling, with a decrease in LVEF from baseline, and the medication was adjusted after hospitalization for exacerbation of CHF. One child died during the follow-up period (Table 4).

**Table 4:** Observation indicators. Comparison of post-treatment data between the dapagliflozin group and the control group.

Variable	dapagliflozin group (*n* = 32)	control group (*n* = 42)	*P*-value
LVEF (%)	43.76 ± 7.58	39.68 ± 8.34	0.032
BNP (pg/mL)	315.00 (193.15–626.25)	511.50 (308.75–859.75)	0.071
NYHA Cardiac Function Classification [n (%)]			0.038
Class I	21 (65.6)	15 (35.7)	-
Class II	10 (31.3)	23 (54.8)	-
Class III	1 (3.1)	4 (9.5)	-

LVEF, left ventricular ejection fraction; BNP, B-type natriuretic peptide; NYHA, New York Heart Association.

Regarding drug-related adverse events, three children developed urinary tract infections during the administration of dapagliflozin, all of which were cured by oral antibiotic therapy (cefdinir for 7 days). In addition, three children developed hypotension, including one who reported dizziness, which improved after dose reduction of sacubitril-valsartan to 2 mg/kg/d. No other adverse reactions, including hypoglycemia or liver and kidney function impairment, were observed at the final follow-up.

## 4 Discussion

SGLT2i, a class of oral hypoglycemic agents, has become a rapidly emerging therapeutic option in the field of type II diabetes since its introduction (10, 11). Surprisingly, this class of drugs has demonstrated cardiovascular event benefit in several clinical studies of type II diabetes combined with CHF, making it useful in the field of CHF treatment as well. It has been suggested (12, 13) that SGLT2i can increase energy expenditure and free fatty acid oxidation, which in turn increases the level of *β*-hydroxybutyrate, a source of energy for cardiomyocytes, and thus improve energy metabolism in cardiomyocytes and cardiac function.

HFrEF is a common clinical cardiovascular condition, characterized by poor overall prognosis and limited treatment options. In recent years, a number of high-level clinical guidelines in Europe and the United States have fully affirmed the benefits of SGLT2i in HFrEF patients and recommended it as one of the four basic drugs for HFrEF treatment. In August 2023, dapagliflozin was approved for the treatment of HFrEF by the National Medical Products Administration in China. However, few of the above guidelines or clinical studies involve pediatric cases.

In 2022, Newland et al. (14) first reported the use of dapagliflozin in children with dilated cardiomyopathy or single ventricle combined with CHF cases. In that study, a total of 37 children with HFrEF received dapagliflozin at an initial dose of 0.1–0.2 mg/kg/d and a maximum dose of 10 mg/d. The results suggested that dapagliflozin could effectively improve LVEF, BNP, and other clinical indicators in children. In the study, six children developed urinary tract infection and four children developed acute renal impairment, but all of them improved after treatment, suggesting that dapagliflozin was relatively well tolerated by children with HFrEF. Cui Jingyi et al. (15) reported the results of a study of dapagliflozin in children with hereditary nephropathy associated with proteinuria, which also suggested the safety of dapagliflozin in children with hereditary nephropathy in different dose ranges.

With reference to the above results, dapagliflozin at a dose of 0.2 mg/kg/d up to 10 mg/d was administered to children with HFrEF in different weight ranges. At the time of the final follow-up visit, three children developed urinary tract infection during the administration of the drug, which was cured by oral anti-infective medication. Three children developed hypotension, including one with dizziness, which was ameliorated by downward modification of the ARNI drug. The remaining children did not experience any other adverse reactions, including hypoglycemia or hepatic or renal impairment.

Regarding the effectiveness of dapagliflozin in pediatric HFrEF cases, the results of this study suggested that, at 24 (±2) weeks of dapagliflozin application, children had a significant increase in LVEF, a decrease in BNP indexes, and a significant improvement in NYHA cardiac function class compared with baseline levels. Compared with children in the control group, the dapagliflozin group showed a more significant increase in LVEF and a higher degree of improvement in NYHA cardiac function classification. However, in the study, all children received a triple combination of ACEI/ARNI, beta-blocker, and aldosterone receptor antagonist in addition to dapagliflozin. In addition, 11 children in the dapagliflozin group received digoxin and two children underwent cardiac resynchronization therapy, while 12 children in the control group received digoxin and one child underwent cardiac resynchronization therapy. The treatment modalities were not identical between the two groups except for dapagliflozin. Therefore, the improvement in LVEF and BNP could not be attributed to dapagliflozin alone but was more likely to be the result of a combination of drugs and therapeutic measures. It should also be noted that NYHA functional classification was developed for adults, so its applicability in infants and young children is limited, and results should be interpreted with caution. Future studies may consider using age-appropriate tools such as the modified Ross Classification for Heart Failure in Children to better evaluate functional improvement.

Previous studies about the usage of dapagliflozin have mainly focused on reducing the risk of death and hospitalization due to worsening heart failure. In the present study, all 32 children treated with dapagliflozin were alive at the last follow-up and did not require hospitalization for worsening heart failure during the 24 (±2) weeks of follow-up, showing a significant advantage over the control group. The latest guidelines for diagnosis and treatment of acute and chronic heart failure in Europe suggest that dapagliflozin significantly reduces the risk of hospitalization but not cardiovascular death (3).However, our study provided novel insights as a real-world, bicenter cohort in China, focusing specifically on the dosing strategy of dapagliflozin, and the results suggested that dapagliflozin may provide additional benefit in children with HFrEF. However, the small sample size of this study and the fact that the underlying diseases in all cases included only dilated cardiomyopathy and congenital heart disease, with no coronary heart disease or ischemic cardiomyopathy, may explain the discrepancy between the results of the study and the adult guidelines.

Recent national and international studies have further suggested that the therapeutic effect of dapagliflozin in CHF is independent of LVEF. Patients with mildly reduced ejection fraction or preserved ejection fraction can also benefit, contributing to strengthened recommendations for dapagliflozin (1–3). Further follow-up studies are needed to determine whether the same effect is seen in pediatric cases.

In summary, dapagliflozin was associated with improved LVEF, reduced BNP levels, and improved NYHA cardiac functional classification, with no hospitalization or death because of worsening heart failure in children with HFrEF observed during short-term follow-up. The weight-stratified dosing regimen also demonstrated acceptable short-term tolerability. However, the sample size of this study is small and the follow-up period is not long; therefore, we look forward to increasing the sample size to provide further high-grade clinical evidence for dapagliflozin in pediatric heart failure patients.

## Data Availability

The original contributions presented in the study are included in the article/Supplementary Material, further inquiries can be directed to the corresponding author.
